# 

**Published:** 1912-07-27

**Authors:** 


### BUREAU OF INFORMATION.

### Rules for Correspondents.

1. Every letter, on whatever subject, must be accompanied by the coupon to be cut from the back cover (inside page) of The Hospital, current issue, and must contain the name and address of the correspondent with pseudonym for publication if desired.

2. Replies by post cannot be given, save under exceptional circumstances at the Editor's discretion.

3. Letters from Approved Homes in reply to special needs published in the Bureau should state terms and full particulars, and be sent prepaid under cover to the Editor of the Bureau with coupon and name enclosed for identification.

4. Proprietors of homes which have not yet been entered on the List of Approved Homes, but have spare accommodation likely to suit special needs, are invited to write for an application form for registration. The fee for registration is 5s.

5. Special needs of every description relating to those who are sick or in difficulties are made known in these columns free of charge.

6. All communications to be addressed to The Editor of The Hospital, 28 Southampton Street, Strand, London, W.C., and marked "Bureau of Information."

### Needs and Helpers.

### Home for Widow Invalided.

J. E. W., M.R.C.S.—It is a very difficult thing to find a home for a woman of sixty-seven, who has suffered from myocarditis, for the small payment of 5s. a week. However, as she is now able to walk about and look after herself it is possible it might be managed in some country village. If her relations could make the amount up to 7s. 6d. we feel sure we could get the case undertaken. At Nazareth House, Hammersmith, cases of this description are received free, and are very kindly treated. Write to the General Superior. Write also to Miss Marsh, 165 Adelaide Road, London, N.W.

### Homes Wanted.

### Inexpensive Permanent Home.

Consuelo (114a).—We are very pleased to make it known that you are seeking a nursing home in central or north-west London, where you could be received for a permanency and nursed during occasional attacks of intermittent fever at a payment of a guinea and a half a week. As you are able, if desired, to furnish your own bedroom, and bring your own linen and plate, there should be no difficulty in your finding the right niche. Prepaid letters from Homes on the List forwarded. Other correspondents must please note Rule 4.

### Training and Employment.

### School for Massage and Electricity.

H. W. B., Secretary.—Many thanks. We are pleased to let "Masseuse" know that there is also a school, to which outside students are admitted, in the above subjects at the Hospital for Epilepsy and Paralysis, Maida Vale, London, W. The existence of these hospital massage schools is too often unknown by those who desire to take up this work. We shall be glad to file the particulars of your course for future reference if you will kindly forward us a syllabus.

### Opening for Male Nurse.

C. B. S.—It is regrettable, but nevertheless a fact, that many of your qualifications will stand you in little stead as a male nurse. By far the most lucrative branch of work for a male nurse with a good knowledge of medical and surgical work and infectious diseases is private nursing. The main difficulty is to form a connection, but well-trained male nurses are much in request and are well paid. Institution posts are not very highly paid, the usual rate for Poor-Law male attendants being £30 a year. We fear that your knowledge of drugs would not count anywhere, as you are not certificated, and we strongly advise you to take the Minor examination of the Pharmaceutical Society, the fee for which is ten guineas, if you are interested in this branch of work. The "slight knowledge of dentistry" would not be an asset in any position of which we have knowledge.

### Medical Course for Women.

Ambitious One.—You are not too old at twenty-one to commence your medical studies, but just right. The course covers five years, and costs from £140 to £160, according to the qualification you aim at. Write for particulars to the Secretary of the London School of Medicine for Women, the Royal Free Hospital, Gray's Inn Road, London, W.C. If the course of study is carried out in Scotland the fees are rather less. For particulars apply to Queen Margaret College School of Medicine for Women, Kelvinside, Glasgow.

### Hospital Construction.

### A Model Mortuary.

Voltaic.—We are glad to inform you that articles on "The Hospital Mortuary" will shortly appear in The Hospital. If you wish to see two good types of mortuary, one for a cottage and one for a larger hospital, you had better make a point of seeing those of the Cirencester Cottage Hospital and of the Coventry and Warwickshire Hospital. We are sure that any matron who is happy enough to have a really well-equipped mortuary attached to her hospital will readily respond to your wish to have a photograph with particulars. We will forward replies. Both letters received.

### Municipal Medicine.

### Treatment of Deformities in Children.

Remedial Gymnast.—The school clinics in contemplation by the County Councils for the treatment of scoliosis and other deformities in children are still in a very inchoate condition, and are not likely to take definite shape before next year. It is impossible to say at present whether "the physical instructress employed by the County Council to give Swedish drill in the secondary schools will be employed for the above work, or whether the post will be filled by a person more specially qualified to undertake curative treatment." Probably school nurses will be employed under the orthopædic surgeon. The appointments will be advertised in the press, and the required qualifications will then be fully set out.

### Medical Terms.

### The Prevalence of Appendicitis.

R. T., Tettenhall.—Appendicitis is not a new disease, though apparently more prevalent now than formerly. It used to be included under the term "perityphlitis," meaning inflammation connected with the cæcum or blind portion of the large intestine. Being now better understood it is more promptly recognised. No specific germ has hitherto been found associated with this disease among the virulent micro-organisms which provoke the bad symptoms, but research in that direction is steadily proceeding. See The Hospital, July 13, p. 386.

### Addresses Wanted.

### Student Christian Movement.

East Indian.—Write to the Rev. Tissington Tatlow, Annandale, North End Road, Golder's Green, London, for information relating to this organisation. The Conference on the Christian Education of Women in the East, to which you allude, will be held at Oxford, September 4-10. Many of the officers of the above movement will take part in it, though it is not held directly under their auspices. The Student Movement is making most encouraging progress at the universities and in colleges for higher education all over the world, and now includes over 100,000 students in twenty-eight different countries.

### Approved Homes.

Note.—*No Home is added to the List without direct medical and other testimony to its good management.*

### For Nerve Cases near London.

Lyndhurst Lodge, Whitehorse Lane, South Norwood, London, S.E.—Proprietors, Mr. and Mrs. Aldridge, trained mental nurses. For slight mental, feeble, paralytic, epileptic or nerve cases. Two acres of wooded ground, three hundred and fifty feet above sea-level. Croquet, bowls, etc. Private rooms. Terms very moderate.

### For General and Nerve Cases.

Sea View House, Canterbury Road, Herne Bay.—Proprietor, Mrs. Ingle. The home has recently been moved to bright and airy premises facing the sea on the East Cliff. It is well adapted for nerve patients, with whom Mrs. Ingle has had wide experience. Medical and surgical patients received also. Massage, rest cures.

### Letter-Box.

R. W., Clapham.—We must refer you to Rule 4, and shall be pleased to forward your friend a form of application should she desire to be entered on our List.

Yorkshire (10636).—Will you let us know what kind of help is required? We think that the mutual arrangement you propose is not likely to be successful unless you have a maid to do the rough work.

A Nurse at Brighton.—Will you kindly send us the name of some friend in Brighton to whom we could refer before mentioning your kind offer?

**The leaflet containing particulars of the Bureau is now ready and will be forwarded free on application.**

### Gifts and Bequests.

Miss Eleanor Vause Walker has bequeathed £1,500 each to the Lebanon Hospital for the Insane and to the Friends' Retreat Hospital for the Insane, York.

Mr. Thomas Marples, of Sheffield, has bequeathed £1,000 each to the Sheffield Royal Infirmary, the Sheffield Royal Hospital, and the Jessop Hospital for Women.

King Edward's Hospital Fund for London has received at the Bank of England the following annual subscriptions :—Lord Iveagh, £500; Messrs. Coutts and Co., £250; Messrs. Glyn, Mills, Currie and Co., £250; Mr. Cecil H. Oliverson, £250; the Vintners' Company, £105; the Duke of Westminster, £100.

### Appointments.

Miss Sara O'Flynn, M.B., Ch.B., has been appointed junior obstetric assistant to the Royal Free Hospital.

On the appointment of Sir William Bennett, K.C.V.O., F.R.C.S., as consulting surgeon of the Seamen's Hospital Society, Mr. C. C. Choyce, F.R.C.S., has been promoted from the junior to the senior surgical staff and Mr. Percival P. Cole, F.R.C.S., has been appointed assistant surgeon to the *Dreadnought*.

Dr. T. W. Eden having resigned the office of physician to in-patients at Queen Charlotte's Hospital, the vacancy on the in-patient staff thus created has been filled by the election of Dr. R. D. Maxwell. Dr. J. H. Drysdale has been elected non-obstetric physician, in succession to Dr. W. P. Herringham, resigned.

### The Editor's Letter-Box.

### Queen Alexandra and King Edward Memorials.

*To the Editor of* The Hospital.

### The Week's Novelties.

### PURDUE'S FLUID SULPHUR.

The use of sulphur-baths for the treatment of many ailments—not only for actual skin diseases, but for some of the constitutional affections—has long been recognised as a valuable therapeutic measure. But the difficulties associated with the satisfactory application of sulphur in this way to a large extent have prevented its extended use. There has now been placed upon the market a liquid form of sulphur, which makes the preparation of a sulphur bath a matter of very much greater simplicity. Messrs. Purdue and Co., of Bermondsey Street, S.E., are the manufacturers of this fluid sulphur, which is to be added direct to a bath of hot water in certain proportions. The fluid is clear and stable, and is manufactured from pure sublimed sulphur, and enables anyone to prepare at small cost of either time or money a sulphur bath which is a very satisfactory imitation of that obtained from a natural thermal sulphur spring. Of the utility of sulphur baths in toning and disinfecting the skin there is no question, and under proper supervision a course of such baths—now so easily attainable—is likely to prove of great benefit to the system.

